# Simultaneous Total Hip Arthroplasty and Plate Fixation for Ipsilateral Subtrochanteric Fracture Following Femoral Neck Osteosynthesis

**DOI:** 10.1155/cro/6505464

**Published:** 2025-12-22

**Authors:** Hirokazu Takai, Ryota Moroi, Masato Shiroma, Kensuke Miyazaki, Shingo Hama, Seiko Takai, Chihiro Hayai, Tomoki Takahashi

**Affiliations:** ^1^ Department of Orthopaedic Surgery, Kumamoto Kinoh Hospital, Kumamoto, Japan, juryo.or.jp; ^2^ Image Diagnostic Center, Kumamoto Kinoh Hospital, Kumamoto, Japan, juryo.or.jp

**Keywords:** complications, femoral neck fracture, insufficiency stress fracture, ORIF (open reduction and internal fixation), subtrochanteric fracture, THA (total hip arthroplasty)

## Abstract

**Background:**

Subtrochanteric fracture is a recognized complication following osteosynthesis for femoral neck fractures, typically occurring within 1–2 months postoperatively. At the time of onset, incomplete healing of the femoral neck fracture often results in a complex fracture configuration that poses challenges for management.

**Case Presentation:**

A 60‐year‐old woman with schizophrenia sustained a Garden Type I femoral neck fracture after a fall and underwent osteosynthesis with three sliding hip screws. Three months later, she developed marked hip pain without any new trauma. Radiographs revealed a displaced ipsilateral subtrochanteric fracture originating from the distal screw insertion site. The patient was treated with simultaneous open reduction and internal fixation (ORIF) using a polyaxial locking plate and total hip arthroplasty (THA). The postoperative course was uneventful, and the patient regained independent ambulation.

**Conclusion:**

Although intramedullary nailing remains a common option for such fractures, selecting the optimal treatment can be challenging, particularly when fixation stability and femoral head viability are in question. In selected middle‐aged or elderly patients, combining joint replacement with fracture fixation can provide both reliable mechanical stability and early functional recovery. This case underscores the importance of considering simultaneous arthroplasty and plate fixation in patients with poor bone quality, incomplete femoral neck healing, or a high risk of avascular necrosis, when revision osteosynthesis alone cannot provide sufficient mechanical stability.

## 1. Introduction

Screw fixation for femoral neck fractures is widely performed as a standard treatment [1, 2]; however, subtrochanteric fracture remains a recognized postoperative complication. Many of these fractures occur within approximately 1–2 months postoperatively [3–7], at which point the femoral neck fracture has not yet achieved bone union, resulting in a complex fracture state. Since both the femoral neck fracture and subtrochanteric fracture must be treated simultaneously, intramedullary nails are often used. However, complications such as nonunion and osteonecrosis of the femoral head make treatment challenging.

In this case report, we present a patient with an ipsilateral subtrochanteric fracture following femoral neck osteosynthesis who was successfully treated with simultaneous open reduction and internal fixation (ORIF) using a polyaxial locking plate and total hip arthroplasty (THA). This combined approach resulted in a favorable postoperative outcome.

## 2. Case Presentation

**Figure 1:**
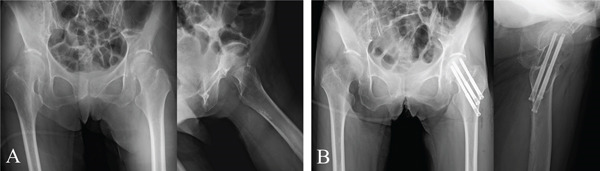
Radiographs of the initial surgery for a left femoral neck fracture. (A) Preoperative image showing a valgus‐impacted Garden I fracture. (B) Postoperative image showing fixation with three sliding screws.

A 60‐year‐old woman (height, 162.5 cm; weight, 44 kg; body mass index, 16.7 kg/m^2^) with schizophrenia was hospitalized in a psychiatric facility when she sustained a femoral neck fracture (Garden Type I) following a fall (Figure 1A).

On the fourth day after injury, she underwent percutaneous internal fixation using three sliding hip screws (Prima Hip Screw, Japan Medical Dynamic Marketing, Tokyo, Japan) (Figure 1B).

Moderate femoral neck shortening was observed, and mild hip pain persisted for 2 months postoperatively, with delayed gait recovery.

Computed tomography (CT) at that time showed no signs of an impending subtrochanteric fracture. Her femoral neck bone mineral density was 0.458 g/cm^2^ (*T*‐score −3.0). She was receiving medical treatment with eldecalcitol and a selective estrogen receptor modulator, and weight bearing was allowed as tolerated according to pain.

**Figure 2:**
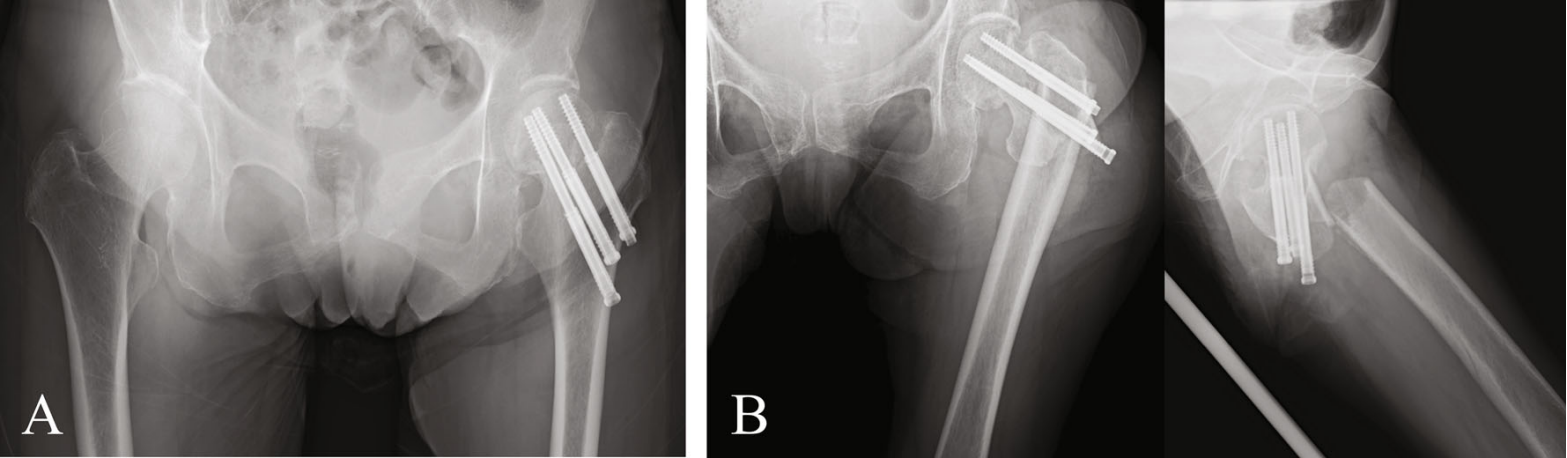
Radiographs before and after the onset of the ipsilateral subtrochanteric fracture. (A) The image obtained immediately before fracture onset demonstrates moderate femoral neck shortening, suggesting delayed union or nonunion. (B) Radiograph obtained 3 months after osteosynthesis shows a displaced subtrochanteric fracture.

Three months postoperatively, she developed severe hip pain without any new trauma or fall. Initial radiographs did not reveal a fracture (Figure 2A), but pain worsened the following day, prompting repeat imaging that demonstrated a displaced subtrochanteric fracture originating from the distal screw insertion site (Figure 2B). The fracture was classified as AO/OTA 32‐A3.1.

**Figure 3:**
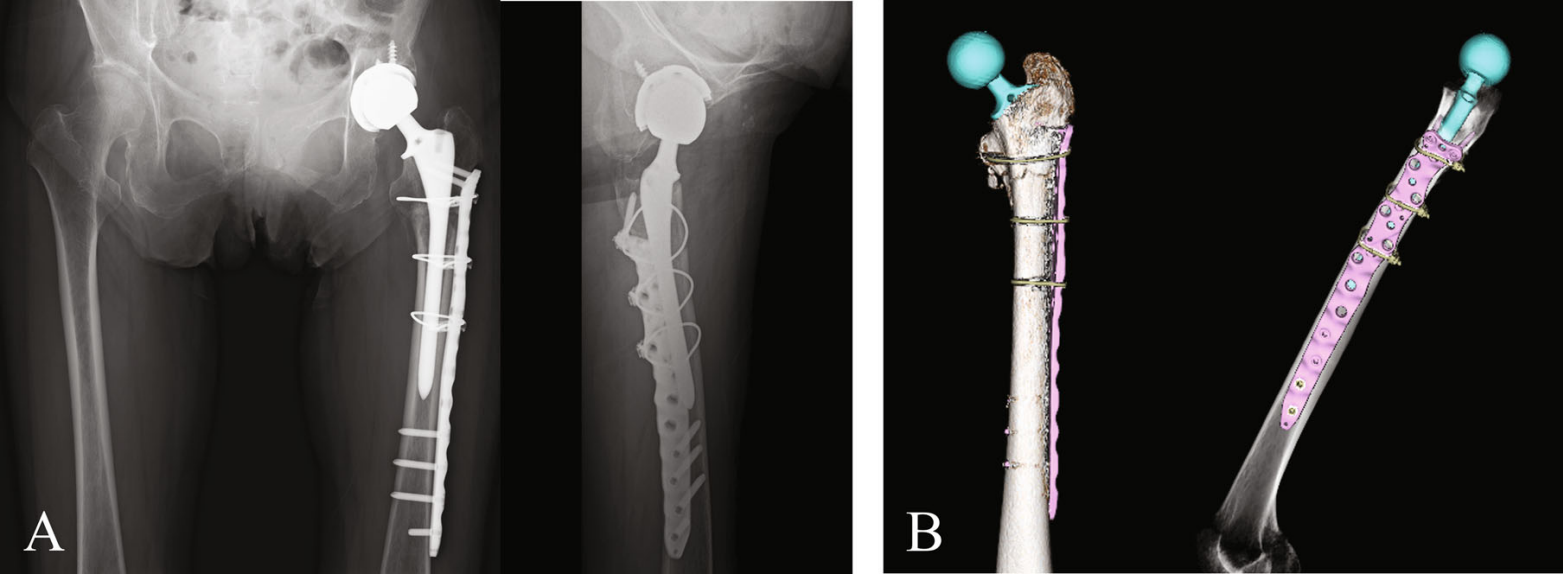
Postoperative radiograph and CT scans. (A) Radiograph showing total hip arthroplasty with a polyaxial locking plate. (B) CT image demonstrating solid fixation with two sufficiently long proximal screws placed without interference with the prosthetic stem.

Under general anesthesia, ORIF was performed using a polyaxial locking plate system designed for periprosthetic femoral fractures (Non‐Contact Bridging Proximal Femur Plate for Periprosthetic Fracture [NCB‐PF‐PP], Zimmer Biomet, Winterthur, Switzerland). THA was then performed via a posterior approach using a fully plasma‐sprayed, distal fixation, uncemented straight stem (Arcos, Zimmer Biomet, Warsaw, IN, United States) (Figure 3).

The postoperative course was uneventful, with no complications such as infection, dislocation, or nonunion. Radiographic bone union was confirmed, and the patient regained independent ambulation.

## 3. Discussion

Subtrochanteric fracture after femoral neck fixation is a rare but serious complication, with a reported incidence ranging from 0.5% to 3% [3–10]. Contributing factors include distal screw insertion beyond the lesser trochanter [3, 6, 9], repeated drilling [3, 7], and screw placement in the distal metaphyseal region [4, 7].

In the present case, although screws were inserted without guidewire reinsertion, the most distal screw was positioned near the lower margin of the lesser trochanter, likely acting as a stress riser that precipitated the subsequent fracture. Importantly, the fracture occurred in the absence of any new trauma or fall, suggesting that accumulated mechanical stress alone led to failure.

Finite element studies have also shown that stress concentration around screw holes contributes to cortical weakening and subsequent fracture risk [11].

The delayed onset—3 months postoperatively—suggests progressive cortical stress accumulation around the screw hole [12], compounded by incomplete remodeling of the femoral neck. Such cases can therefore also be regarded as insufficiency stress fractures, particularly in patients with osteoporosis or reduced bone turnover, and awareness of this mechanism is essential for postoperative monitoring and early detection.

### 3.1. Surgical Challenges and Approach

A characteristic feature of subtrochanteric fractures following femoral neck osteosynthesis is that the femoral neck fracture has often not yet achieved bone union, resulting in a complex fracture state. Screws already placed for femoral neck fixation further complicate treatment [7, 9, 10]. While intramedullary nails are typically used, they carry risks of malreduction, nonunion, and osteonecrosis of the femoral head [13–16].

In our case, simultaneous ORIF and THA were chosen because persistent hip pain and femoral neck shortening suggested nonunion or early avascular necrosis, making long femoral nail fixation unsuitable [17–19]. Blomfeldt et al. reported that arthroplasty can result in lower complication rates and improved postoperative function in such cases [20]. Several reports have also demonstrated favorable outcomes of cementless THA for failed fixation or subtrochanteric fractures [21–23].

### 3.2. Surgical Technique

To stabilize the subtrochanteric fracture, plate fixation combined with supplementary cable wiring was performed. Before fracture reduction, drill holes were made in the lesser trochanter and distal femoral diaphysis, through which cable wires were passed in advance to facilitate later fixation. After achieving Blomfeldt′s anatomical reduction, the cables were tightened, and temporary fixation was obtained using Kirschner wires, followed by definitive fixation with a locking plate.

**Figure 4:**
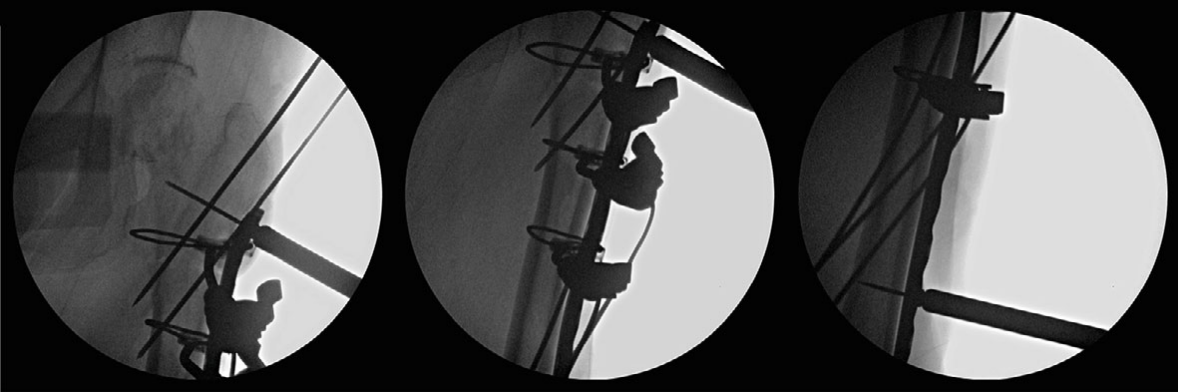
Intraoperative fluoroscopy. Before reducing the fracture, pass wires through predrilled holes in the lesser trochanter and distal femoral diaphysis. After reduction, temporarily fix the fracture with Kirschner wires and then apply a plate for definitive stabilization.

Screws were inserted distal to the planned tip of the femoral stem. The proximal screws were placed anterior and posterior to the stem, along the intertrochanteric ridge, ensuring sufficient length without interference with the prosthesis (Figure 4). This configuration provided rigid fixation of the fracture, allowing THA to be performed under stable conditions, similar to a primary THA.

A fully plasma‐sprayed, distal‐fixation, uncemented straight titanium stem (Arcos, Zimmer Biomet, Warsaw, IN, United States) was used, providing fixation across both proximal and distal regions. The extensive porous coating promotes excellent bone ingrowth, enabling durable fixation and long‐term stability even in cases of fracture, nonunion, or revision arthroplasty [24, 25].

**Figure 5:**
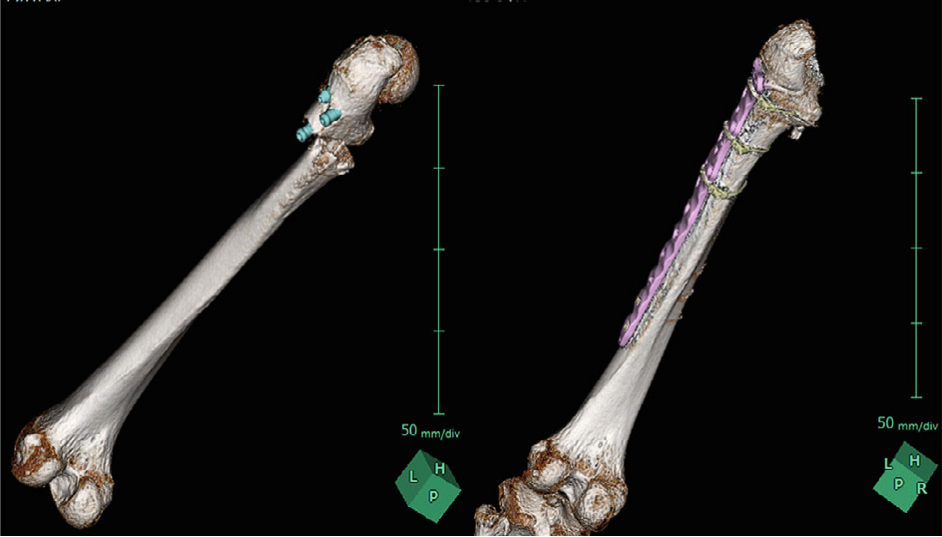
Reconstructed 3D representation of femur positioning for prosthetic stem insertion. Postoperative CT reconstruction illustrates the femoral alignment during stem insertion in the posterior approach. In transverse subtrochanteric fractures, excessive hip flexion and internal rotation can misalign the proximal fragment. Performing osteosynthesis beforehand allows the limb to be maintained in the optimal position for stem insertion.

Postoperative 3D CT reconstruction (Figure 5) demonstrates the final configuration of the plate and cable wires. This construct maintained optimal femoral alignment during stem insertion, reduced implant stress, and is expected to lower the risk of stem fatigue failure.

## 4. Conclusion

Simultaneous ORIF with THA can be a viable and durable alternative to intramedullary nailing for ipsilateral subtrochanteric fractures following femoral neck osteosynthesis, especially in middle‐aged or elderly patients with poor bone quality or incomplete femoral neck healing.

This combined approach offers stable fixation, reduces the risk of nonunion and femoral head osteonecrosis, and allows early mobilization and functional recovery.

Moreover, careful postoperative monitoring for stress‐related cortical changes around screw insertion sites is essential to detect early mechanical failure and to prevent catastrophic subtrochanteric fractures in similar patients.

## Ethics Statement

This report was conducted in compliance with the tenets of the Declaration of Helsinki. Ethical approval was not required by the authors′ affiliated institution, as this case report did not involve experimental interventions or research procedures.

## Consent

Written informed consent was obtained from the patient for the publication of this case report and the accompanying images.

## Conflicts of Interest

The authors declare no conflicts of interest.

## Funding

No funding was received for this manuscript.

## Data Availability

The data that support the findings of this study are available from the corresponding author upon reasonable request.
